# The Functional SNPs in the 5’ Regulatory Region of the Porcine *PPARD* Gene Have Significant Association with Fat Deposition Traits

**DOI:** 10.1371/journal.pone.0143734

**Published:** 2015-11-24

**Authors:** Yunxia Zhang, Tengsen Gao, Shanyao Hu, Bin Lin, Dechao Yan, Zaiyan Xu, Zijun Zhang, Yuanliang Mao, Huimin Mao, Litong Wang, Guoshui Wang, Yuanzhu Xiong, Bo Zuo

**Affiliations:** 1 Key Laboratory of Swine Genetics and Breeding, Ministry of Agriculture and Key Lab of Agricultural Animal Genetics and Breeding, Ministry of Education, College of Animal Science and Veterinary Medicine, Huazhong Agricultural University, Wuhan, P. R. China; 2 The Tianpeng Group, Jiangshan, Zhejiang, P. R. China; Guangzhou Institute of Biomedicine and Health, CHINA

## Abstract

Peroxisome proliferator-activated receptor delta (*PPARD*) is a key regulator of lipid metabolism, insulin sensitivity, cell proliferation and differentiation. In this study, we identified two Single Nucleotide Polymorphisms (SNPs, g.1015 A>G and g.1018 T>C) constituting four haplotypes (GT, GC, AC and AT) in the 5’ regulatory region of porcine *PPARD* gene. Functional analysis of the four haplotypes showed that the transcriptional activity of the *PPARD* promoter fragment carrying haplotype AC was significantly lower than that of the other haplotypes in 3T3-L1, C2C12 and PK-15 cells, and haplotype AC had the lowest binding capacities to the nuclear extracts. Transcription factor 7-like 2 (TCF7L2) enhanced the transcription activities of promoter fragments of *PPARD* gene carrying haplotypes GT, GC and AT in C2C12 and 3T3-L1 cells, and increased the protein expression of *PPARD* gene in C2C12 myoblasts. TCF7L2 differentially bound to the four haplotypes, and the binding capacity of TCF7L2 to haplotype AC was the lowest. There were significant associations between -655A/G and fat deposition traits in three pig populations including the Large White × Meishan F_2_ pigs, France and American Large White pigs. Pigs with genotype *GG* had significantly higher expression of *PPARD* at both mRNA and protein level than those with genotype *AG*. These results strongly suggested that the SNPs in 5’ regulatory region of *PPARD* genes had significant impact on pig fat deposition traits.

## Introduction

Peroxisome proliferator-activated receptors (PPARs) are lipid-activated nuclear receptors belonging to the nuclear hormone receptor superfamily [1]. Ligand-activated PPARs form heterodimers with retinoic X receptors (RXRs) which bind to PPAR response elements (PPREs) and positively regulate transcription of target genes [2]. PPARs are involved in a number of biological processes, including lipid metabolism [3], insulin sensitivity, inflammation [4], cell proliferation and/or differentiation [5]. *PPARD* gene is widely expressed in the tissues including liver, hearts, adrenal, intestine and adipose in mice and rats [6, 7]. It enhances gene transcriptions that are involved in the fatty acid transport, oxidation, energy uncoupling, mitochondrial respiration and thermogenesis [1, 8]. *PPARD* inhibits the onset of oxidative stress-induced apoptosis in H9c2 cells [2], cell proliferation in keratinocytes [5], vascular smooth muscle cells [9], lung fibroblasts [10], and cardiac fibroblasts [11, 12]. In preadipocytes, *PPARD* gene begins to be expressed during the early periods of induced differentiation *in vitro* [13], playing important roles in the regulation of adipogenesis by fatty acid [14]. *PPARD*-deficient mice showed multiple developmental and homeostatic abnormalities, including placental defects causing frequent embryonic lethality, decreased adipose mass, altered skin inflammatory responses, and impaired wound healing [15–17]. In human, DNA variations within the *PPARD* genomic sequence are significantly associated with body mass index, high-density lipoprotein cholesterol, leptin, skeletal muscle glucose uptake, homeostasis model assessment of insulin resistance (HOMA-IR), adiposity measures or fasting serum lipids and height [18–21].

We are interested in studying pig fat deposition because it plays important roles in animal agriculture and can be used as biomedical model for human obesity. Pig carcass composition such as backfat thickness and lean meat percentage are controlled by polygenes with pleiotropic effects. Identification of these polygenes or linked markers is necessary for understanding the genetic basis of carcass traits and the application of marker assisted selection (MAS) in breeding programs [22]. A significant quantitative trait loci (QTL) for backfat thickness (BFT) has been consistently mapped to *sus scrofa* chromosomes 7 (SSC7) p1.1-q1.4 in several pig populations [23–31]. Using the F_2_ resource population derived from the intercross of Large White boars and Meishan dams, we have identified significant QTLs for carcass and meat quality traits on SSC7 [23, 32]. Haplotypes of the porcine *PPARD* gene are associated with backfat thickness [33], and *PPARD* gene also functions in fatty acid metabolism and fat metabolism [34]. Therefore, *PPARD* gene was considered to be a promising positional candidate gene for the fat deposition traits. Previous studies also showed that one missense mutation within the coding sequence of *PPARD* gene was significantly associated with ear size [35, 36]. However, whether and how the genetic variants within upstream regulatory region of *PPARD* gene affected fat deposition traits were still unknown in pigs. In this study, we identified two functional SNPs (GU565976.1: g.1015 A>G and g.1018 T>C) in the 5’ regulatory region of *PPARD* which altered the binding capacity transcription factor TCF7L2 to the promoter region, and found that the SNP g.1015A>G was significantly associated with fat deposition traits in three pig populations.

## Materials Methods

### Animals and trait measurement

All animal procedures were performed according to protocols approved by the Animal Care and Use Committee of Huazhong Agricultural University, Hubei province, P. R. China. Pigs from thirteen pig populations (12 Chinese Bamei pigs, 19 Jianli pigs, 16 Exihei pigs, 44 Meishan pigs, 34 Erhualian pigs, 19 Wannan pigs, 27 Huainan pigs, 31 English Large White pigs, 34 Landrace pigs, 19 Yangxin pigs, 409 American Large White pigs, 710 France Large White pigs, and 274 Large White × Meishan F_2_ pigs) were used to investigate the distribution of allele and genotype frequency of SNP g.1015A>G. The association analyses were conducted in three pig populations including 274 Large White × Meishan F_2_ pigs, 409 American Large White pigs and 710 France Large White pigs. All traits were measured and recorded according to *the Principles and methods of swine testing* [37]. The fat deposition and carcass traits were as following: backfat thickness at shoulder (BFT1, cm), backfat thickness at thorax-waist (BFT2, cm), backfat thickness at buttock (BFT3, cm), average backfat thickness at shoulder, thorax-waist and buttock (ABT, cm), leaf fat wight (LFW, kg), backfat thickness between 6^th^ and 7^th^ ribs (67RIBBF, cm), rib number (RN), carcass length from the first cervical vertebra to anterior border of pubic symphysis (CL1, cm), carcass length from the first thoracic vertebra to anterior border of pubic symphysis (CL2, cm), Internal fat rate (IFR). The live backfat thickness were measured by B-ultrasound machine (ESAOTE, Mylob Touch VET) between 85 kg and 115 kg body weight and then corrected to the backfat thickness at 100 kg body weight according to the national standard of swine performance testing of P. R. China (NY/T822-2004). Sequencing and haplotypes inference were conducted in 90 pigs from seven breeds, including 9 Chinese Bamei pigs, 6 Erhualian pigs, 4 Huainan pigs, 5 Duroc pigs, 20 American Large White pigs, 25 France Large White pigs, and 21 Large White × Meishan F_2_ pigs.

### SNP detection and haplotype inference

In order to detect SNPs in the *PPARD* 5’ regulatory region, we sequenced the promoter region (GenBank accession no. GU565976.1) from five Meishan pigs and five Large White pigs, respectively. PCR products of 544 bp, 568 bp and 757 bp spanning nucleotides − 1415 bp to 464 bp (1880 bp length) were sequenced to detect the SNPs in the 5’ regulatory region. The mix of PCR contained 1.5 mM MgCl_2_. The primers for DNA sequencing, amplification and annealing temperature are listed in S1 Table. The purified PCR products of five Meishan and five Large White pigs were cloned into the pMD18-T vector (TaKaRa, Japan), and sequenced commercially (Sangon, China). Then these sequences were aligned using Clustalw (http://www.ebi.ac.uk/Tools/msa/clustalw2/) to detect the SNPs. Direct sequencing of 568 bp PCR products containing the SNPs (g.1015 A>G and g.1018 T>C) were further conducted to infer the haplotypes. Haplotypes were inferred using Haploview software (Version 4.2) [38].

### Vectors construction of promoter fragments carrying the four haplotypes

**Fig 1:**
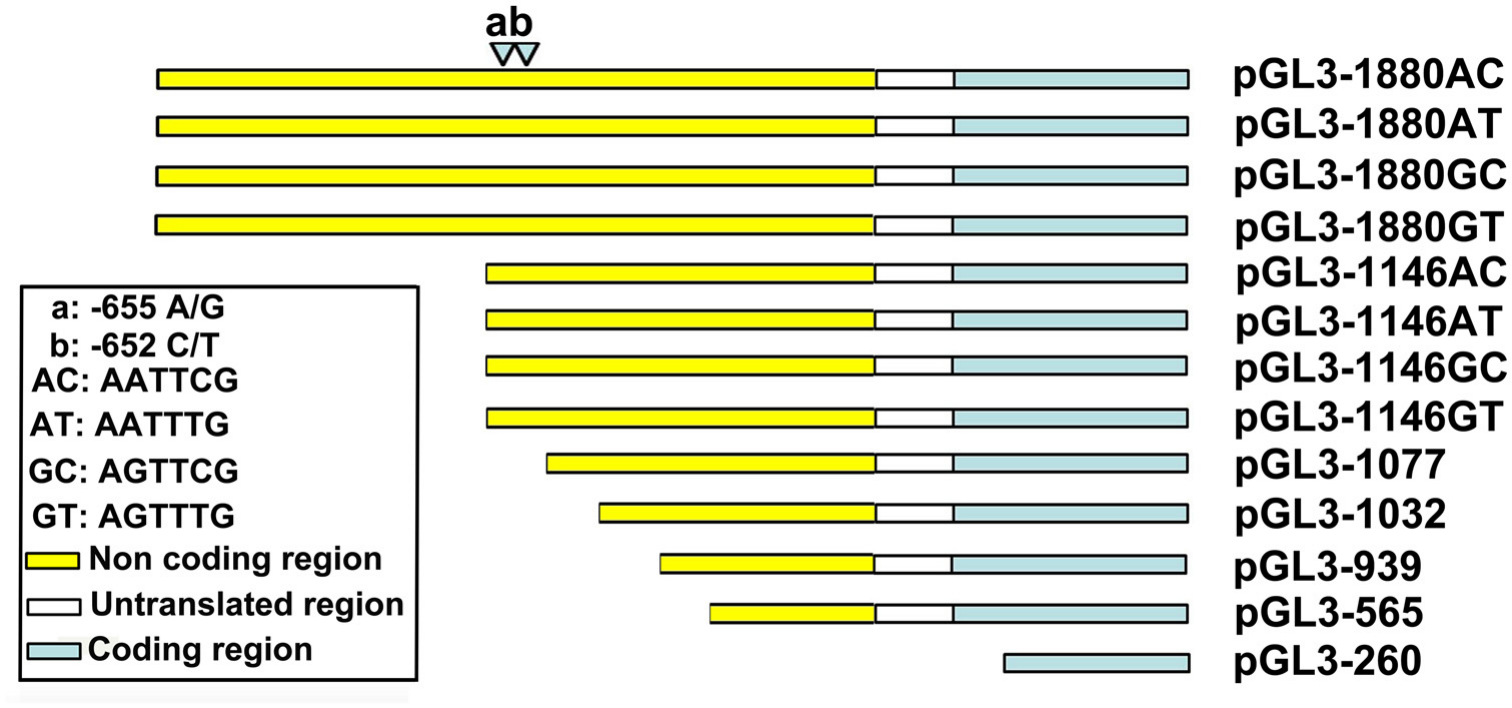
Schematic representation of the deletions of *PPARD* promoter linked with the luciferase gene in vectors. The nucleotides were numbered relative to the transcription start site that was assigned as + 1.

The luciferase reporter gene assays were performed using vector constructs containing the 5’ flanking region of *PPARD* gene: pGL3-1880GT (from − 1415 bp to + 464 bp), pGL3-1146GT (− 681 bp to + 464 bp), pGL3-1077 (− 612 bp to + 464 bp), pGL3-1032 (− 567 bp to + 464 bp), pGL3-939 (− 474 bp to + 464 bp), pGL3-565 (− 100 bp to + 464 bp) and pGL3-260 (+ 205 bp to + 464 bp) (relative to the transcription start site) (Fig 1). The 5’ terminal primer oligonucleotides contained *Kpn*Iand *Sac*Ienzymes sites. The amplified fragments were inserted into the pGL3-Basic vector (Promega, USA). Five deletion fragments of the *PPARD* promoter were generated by PCR using the pGL3-1880GT vector as a template. All primers are listed in S2 Table.

Using the pGL3-1880GT and pGL3-1146GT vectors as templates, two sets of promoter luciferase report gene vectors were constructed by site directed mutagenesis respectively. The vectors included haplotype AC (pGL3-1880AC and pGL3-1146AC), AT (pGL3-1880AT and pGL3-1146AT), GT (pGL3-1880GT and pGL3-1146GT) and GC (pGL3-1880GC and pGL3-1146GC).

The eukaryotic overexpression vector of TCF7L2 (pCDNA3.1-TCF7L2) was constructed. The primer (F: 5’ GGGGTACCGCCACCATGCCGCAGCTGAACGG 3’, R: 5’ GCTCTAGACTATTCTAAAGACTTGGTGACCAGG 3’) was designed according to the GenBank database sequence (NM001142922.1) and used to obtain the CDS (coding sequence) of the swine *TCF7L2* gene. The CDS was digested with restriction enzyme *Kpn*I(forward primer) and *Xba*I(reverse primer), and then ligated into pcDNA3.1 using T4 DNA Ligase (Takara, Japan) to generate pcDNA3.1-TCF7L2 vector.

### Dual luciferase assay

C2C12, 3T3-L1 and PK cells were grown in Dulbecco's Modified Eagle's Medium (DMEM) (Hyclone, USA) supplemented with 10% (v/v) fetal bovine serum (Gibco, Australia), maintained in a 5% CO_2_ incubator at 37°C. Twenty-four hours before transfection, cells were plated onto 24-well plates or 6-well plates. When cells reached 80% confluence, they were transfected with promoter constructs (1 μg) or eukaryotic expression vectors (4 μg) using lipofectamine 2000 (Invitrogen, USA) according to the manufacturer’s instructions.

After transfection for 24 h, cells were lysed in 100 μl of lysis buffer, and firefly and renilla luciferase activities were measured using the Dual-Luciferase Reporter Assay System (Promega, Madison, WI, USA). The luciferase activity was measured using PerkinElmer 2030 Multilabel Reader (PerkinElmer, USA). To normalize the transfection efficiency, the pRL-TK plasmid vector (Promega, USA) was co-transfected with the reporter construct as described above. The experiments were performed in three replicates for each construct and the data were the average of three replicates.

### Quantitative real-time PCR

For tissue expression pattern analysis, nine tissues including heart, liver, spleen, lung, kidney, stomach, intestines, longissimus dorsi (LD), and backfat were sampled from three France Large White pigs. For differential gene expression analysis between different genotypes, muscle and adipose tissues were sampled from three France Large White pigs with genotypes *AG* and *GG*, respectively. RNA samples from different tissues of the France Large White pigs were isolated using the TRIzol reagent (Invitrogen, USA). Reverse transcription of RNA (1 μg) was performed using random primers (Invitrogen, USA) and Moloney Murine Leukemia Virus (M-MLV) Reverse Transcriptase (Invitrogen, USA) according to the manufacturer’s instructions. The primers used to amplify the cDNA corresponding to *PPARD*, *TCF7L2*, and *β-actin* genes are listed in S3 Table. Quantitative RT-PCR was performed on a LightCycler 480 II Real-Time (Roche, Switzerland) using the FastStart DNA Master SYBR Green I reagent (Roche, Switzerland). The gene expression results were normalized with regard to the expression of the *β-actin*. The Ct (2^-ΔΔCt^) method was used to analyze the relative gene expression [39].

### Western blotting

Tissues and cells were lysed in RIPA buffer according to manufacturer’s instruction (Beyotime, China). Protein lysates were heated at 95°C 5min in 5 × Sodium Dodecyl Sulfate (SDS) sample buffer separated by 12% SDS-PAGE (20 μg each lane), then transferred to polyvinylidene fluoride (PVDF) membranes (Millipore, USA) using Mini Trans-Blot Cell (Bio-Rad, USA). After being blocked with 5% non-fat milk for 1.5 h, the membranes were incubated with primary antibodies overnight at 4°C. After washed three times, the membranes were hybridized with secondary antibody for 1 h at 37°C, and washed three times. The targeted proteins were detected using the ImageQuant LAS 4000 mini (GE, USA) according to the manufacturer instructions. Primary antibodies were specific for PPARD (Abcam, USA, ab23673; 1:1000 dilution), TCF7L2 (Abcam, USA, ab32873; 1:1000 dilution), and β-actin (Boster, China, BM0627; 1:1000 dilution). Secondary antibodies were goat anti-mouse IgG-HRP (Santa Cruz, USA, sc-2005; 1:3000 dilution) and goat anti-rabbit IgG-HRP (Santa Cruz, USA, sc-2004; 1:3000 dilution).

### Electrophoretic mobility shift assay (EMSA)

Nuclear extracts from C2C12 myoblasts, 3T3-L1 cells and pig longissimus muscle were prepared by Nuclear Protein Extraction Kit (BestBio, China). The protein concentration was determined by Bicinchoninic Acid assay (Beyotime, China). The double-stranded DNA probes of four haplotypes were prepared by annealing the desired sense and anti-sense oligonucleotides which were 5’ end-labeled with biotin. The DNA binding activity of protein was detected by chemiluminescent EMSA Kit (Beyotime, China). The protein-DNA complexes were analyzed by electrophoresis in 6.0% polyacrylamide gels at 100 V for 1 h in 0.5 TBE running buffer (44.58 mM Tris Base, 44.58 mM Boric Acid, 1.25 mM Na_2_EDTAH_2_O), and then were transferred to a nylon membrane. The dried nylon was visualized using the ECL (Bio-Rad, USA). The bands were detected using the the ImageQuant LAS 4000 mini (GE, USA) according to the manufacturer’s instruction.

### DNA pull-down

The probes for DNA pull-down assay were the same as EMSA assay. The probes were conjugated with M-280 Streptavidin Dynabeads (Invitrogen, USA) in binding buffer (10 mM Tris-HCl, pH 7.5, 50 mM KCl, 1 mM MgCl_2_, 1 mM EDTA, pH 8.0, 1 mM Na3VO4, 5 mM DTT, 5% glycerol, 0.3% NP-40) for 40 min at room temperature. Non-denaturing total proteins were extracted from 3T3-L1cells using Western and IP Cell lysis Buffer (Sangon Biotech, China). The proteins (400 μg) were incubated with unconjugated Dynabeads for 2 h at 4°C using Dynal MPC-S magnetic particle concentrator (Dynal Biotech, Norway), and the non-specific binding proteins were removed. The supernatant (supernatantI) was collected for binding reaction. The probes were incubated with the Dynabeads for 5 h at 4°C, and then the Dynabeads-probes complexes were washed three times with lysis buffer and incubated with supernatant I for 2 h at 4°C in the presence of 25 μg/ml poly (dI:dC) which could prevent the nonspecific binding of protein with DNA. The precipitates (Dynabeads-probes-protein) were washed with lysis buffer, and then were eluted in SDS sample buffer. The supernatantII was collected for β-actin detection. The precipitates and supernatant were assayed by western blotting.

### SNP genotyping and statistical analysis

The SNP (g.1015A>G) was genotyped by PCR-RFLP using *EcoR*I restriction enzyme. 8.5 μl PCR products were digested with 5 U *EcoR*I restriction enzyme (Fermentas, Canada) for 6 h at 37°C, and then were separated by electrophoresis on a 1.5% agarose gel with ethidium bromide in 1× TAE buffer.

The association of SNP genotypes with fat deposition and carcass traits in Large White × Meishan F_2_ pigs, France, and American Large White pigs were conducted with the general linear model (GLM) procedure of SAS version 8.0 (SAS Institute, Cary, NC, 2000). Both additive and dominance effects were estimated using the REG procedure of SAS version 8.0. The data are presented as mean ± S.D., and statistical significance level was set at *p* < 0.05.

The model of association analysis in 274 Large White × Meishan F_2_ pigs was as following:

$$T_{ijkl}\;=\;\mu \;+\;S_{i}\;+\;Y_{j}\;+\;G_{k}\;+\;F_{l}\;+\;b_{ijkl}X_{ijkl}\;+\;e_{ijkl},$$

Where, *T*_*ijkl*_ is the observed values of a given trait; *μ* is the overall mean; *S*_*i*_ is effect of sex (*i* = 1 for male or 2 for female); *Y*_*j*_ is the effect of year (*j* = 1 for year 2000 or 2 for year 2003); *G*_*k*_ is the effect of genotype (*k* = *AA*, *AG* and *GG*); *F*_*l*_ is the effect of family (*l* = 37); *b*_*ijkl*_ is the regression coefficient of the slaughter weight for carcass traits, *X*_*ijkl*_ is the slaughter weight; *e*_*ijkl*_ is the random residual.

The model of association analysis in 710 France Large White pigs and 409 American Large White pigs was as following:

$$T_{ijkl}\;=\;\mu \;+\;S_{i}\;+\;F_{j}\;+\;G_{k}\;+\;B_{l}\;+\;e_{ijkl},$$

Where, *T*_*ijkl*_ is the corrected backfat thickness at 100 kg live weight; *G*_*k*_ is the effect of genotype (*k* = *AA*, *AG* and *GG*); *S*_*i*_ is the effect of sex (*i* = 1 for male or 2 for female); *F*_*j*_ is effect of family (*j* = 17 for France Large White and *j* = 20 for American Large White); *B*_*l*_ is the effect of batch (*l* = 20 for France Large White and *l* = 8 for American Large White); *e*_*ijkl*_ is the random residual.

## Results

### Identification of two SNPs in the upstream of the 5’ regulatory region of *PPARD* gene

Two SNPs, g.1015A>G and g.1018 T>C (GenBank accession no. GU565976.1) were detected at the upstream − 655 bp (A/G) and − 652 bp (T/C) (relative to the transcription start site + 1) of the 5’regulatory region of *PPARD* gene (Fig 1). The corresponding variation ID of two SNPs in the SNP database (http://www.ncbi.nlm.nih.gov/SNP) was rs80912557 and rs80964726, respectively. The haplotypes of two SNPs were inferred in 90 pigs randomly selected from seven breeds including Bamei, Erhualian, Huainan, Duroc, American Larger White, France Larger White, and Large White × Meishan F_2_ pigs. In these pig populations, the two SNPs were in strong linkage disequilibrium (D = 0.925, R^2^ = 0.633) and the frequency of haplotypes GT, GC, AC and AT was 52.9%, 8.9%, 36.7% and 1.6%, respectively. In American Large White pigs, the frequency of haplotypes GT, GC, AC and AT was 36.7%, 6.7%, 57.7%, and 0%, respectively. In France Large White pigs, the frequency of haplotypes GT, GC, AC and AT was 86.7%, 10.0%, 3.3%, and 0%, respectively. In Large White × Meishan F_2_ pigs, the frequency of haplotypes GT, GC, AC and AT was 32.1%, 25.9%, 42.9%, and 0%, respectively. The results suggested that the allele *A* at rs80912557 of the *PPARD* was mostly linked with allele *C* at rs80964726, and allele *G* at rs80912557 was linked with allele *T* or *C* at rs80964726 in the pig population studied.

### The SNPs in the promoter of *PPARD* gene significantly affected its transcriptional activity

**Fig 2:**
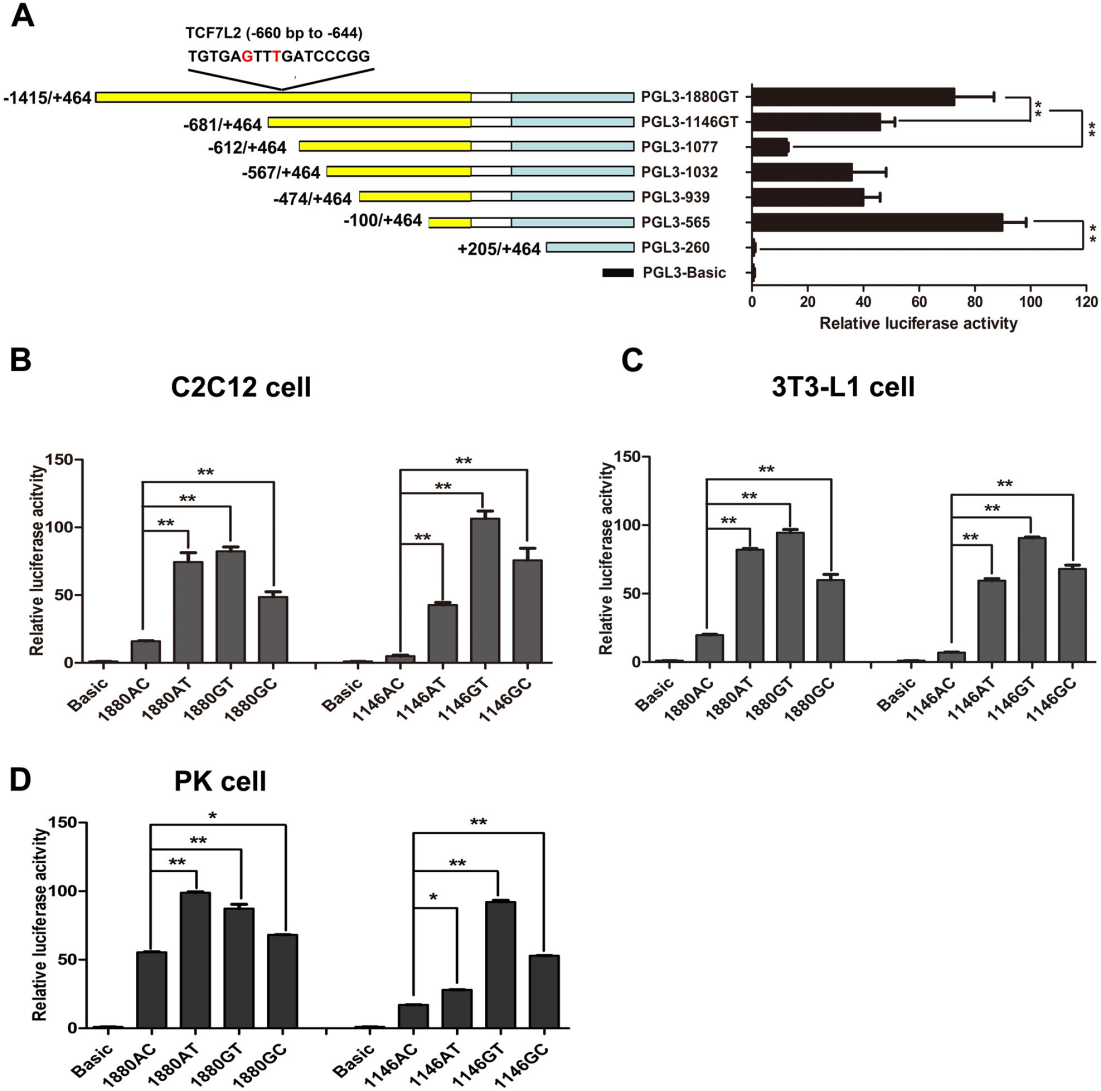
Transcriptional activities analysis of deletion constructs of *PPARD* promoter carrying the four haplotypes. (A) Transcriptional activities of a series of deletion fragments determined by luciferase assay in C2C12 myoblasts. Left panel, schematic representation of the deleted fragments linked with the luciferase gene in the pGL3 vector. The nucleotides were numbered relative to the transcription start site that was assigned as + 1. Right panel, the relative activities of a series of deletion fragments of pGL3-1880GT vector determined by luciferase assay. (B)(C)(D) The relative activities of a series of deletion fragments of *PPARD* promoter carrying the different haplotypes in C2C12, 3T3-L1, and PK cells. Error bar represents mean ± S.D. (three independent replicates per group). Asterisk (*) and (**) represent the significance level at *P* < 0.05 and *P* < 0.01, respectively (The same below).

The bioinformatics prediction of transcription factor binding sites showed that five transcription factors could bind to the segment containing the two SNPs, and the prediction scores of the binding capacity of transcription factors with different haplotype promoter fragments are listed in S4 Table. According to the bioinformatics prediction of transcription factor binding sites by TESS and Promoter 2.0 software, we constructed a series of deletion fragments by fusing different fragments of the 5’ flanking region to luciferase reporter vector pGL3-basic (Fig 1). All constructs were transfected into C2C12 myoblasts and their promoter activities were examined. The luciferase activity of the promoter fragments PGL3-260 (+ 205 bp to + 464 bp) was the lowest in all the luciferase activities (Fig 2A), suggesting that this region between nucleotides − 100 bp to + 205 bp was the core promoter region. The transcription activity of the promoter fragments PGL3-1077 (− 612 bp to + 464 bp) was lower than that of the other two promoter fragments PGL3-1880GT (− 1415 bp to + 464 bp), and PGL3-1146GT (− 681 bp to + 464 bp). The results showed that the promoter fragments from position − 1415 bp to − 612 bp contained the potential positive regulatory elements. To determine the effects of the SNPs on the promoter transcriptional activities, we designed and studied two sets of promoter luciferase report gene vectors carrying the four haplotypes constructed by site-directed mutagenesis using pGL3-1146GT and pGL3-1880GT as templates. The promoter vectors were transfected into C2C12, 3T3-L1, and PK cells, respectively. In C2C12 myoblasts, the transcriptional activity driven by pGL3-1146AC decreased 8.5-fold, 12.9-fold, and 9.7-fold compared with that driven by pGL3-1146AT, pGL3-1146GT and pGL3-1146GC, respectively (Fig 2B). The same trends were found in 3T3-L1 cells and PK cells. The luciferase expression level driven by pGL3-1880AC was also the lowest among the four haplotype constructs (Fig 2C and 2D). The results indicated that the SNPs in the promoter of *PPARD* gene significantly affected its transcription activities. The transcription activities of *PPARD* gene carrying haplotype AC were the lowest and these of haplotype GT the highest.

### Potential transcription factors binding to the promoter fragments carrying the four haplotypes

**Fig 3:**
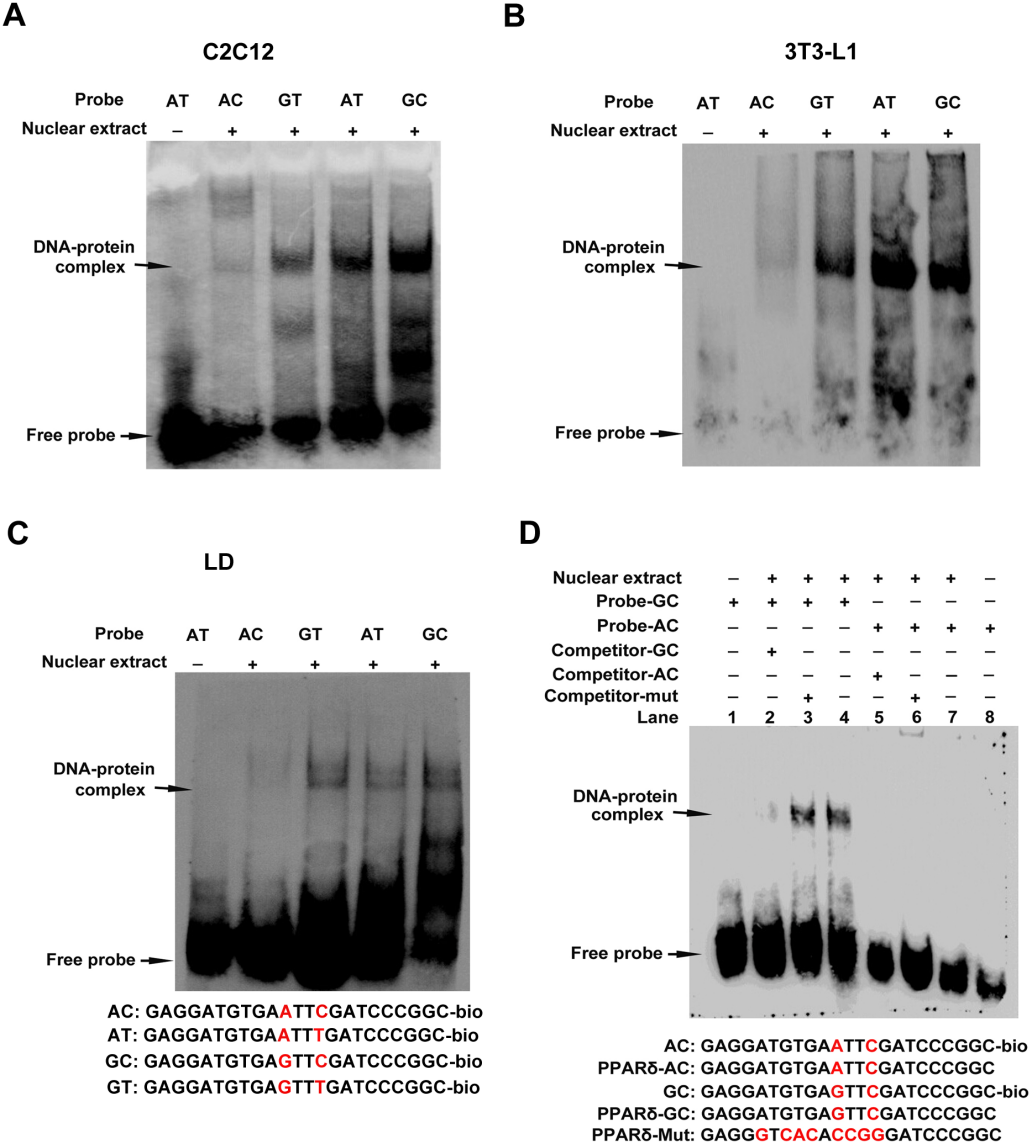
Nuclear extracts differentially bind to the promoter fragments carrying the four haplotypes. (A) (B) (C) EMSA results showing the binding capacities of four haplotypes to the nuclear extracts in C2C12 myoblasts, 3T3-L1 cells, and porcine LD muscle. (D) The EMSA results showing the binding capacities of nuclear extracts to the promoter fragments with haplotypes AC and GC, Lane 1 and Lane 8 were negative control; Lane 2–4 and 5–7 in turn were sample reactions, mutation competitive reactions, and cold competitive reactions for AC and GC reaction groups, respectively. The probes were incubated with nuclear extracts in the absence or presence of 1-fold excess of various competitor probes (mutant or non-labeled probe). The specific DNA-protein complex bands were indicated by arrows. The sequences of various probes were shown under the panel.

We used genomatrix (http://www.genomatrix.de/) to investigate the potential transcription factors in the promoter region harboring the SNPs identified. The results showed that there were potential ETS1, MEL1, Foxh1, POU2F1, and TCF7L2 binding sites in the promoter region, and the binding capacities of Foxh1, POU2F1 and TCF7L2 to the promoter changed when the SNPs were introduced to the DNA sequences (S4 Table). EMSA was performed to detect the binding capacities of different haplotypes to the nuclear extracts in C2C12 myoblasts, 3T3-L1 cells and pig LD muscle. The results of EMSA showed that the binding capacity of haplotype AC to the nuclear extracts was the weakest in C2C12 myoblasts, 3T3-L1 cells and pig LD muscle (Fig 3A, 3B and 3C), confirming that there are potential transcription factors differentially binding to the promoter fragments carrying the different haplotypes. EMSA was further conducted in C2C12 myoblasts with the probes of haplotypes AC and GC which showed the largest difference in the binding capacities (Fig 3D). The incubation of nuclear extracts of C2C12 myoblasts with probe GC formed a DNA-protein complex. The complex became weaker with 1 × cold probes in the mixture. The complex did not change in the mutation cold probe reaction. By contrast, the incubation of nuclear extracts of C2C12 myoblasts with probe AC did not form the DNA-protein complex. These results demonstrated that transcription factors can bind to the DNA sequence with haplotype AT, GT and GC, but not to haplotype AC.

### The *PPARD* gene SNPs showed different interactions with transcription factor TCF7L2

**Fig 4:**
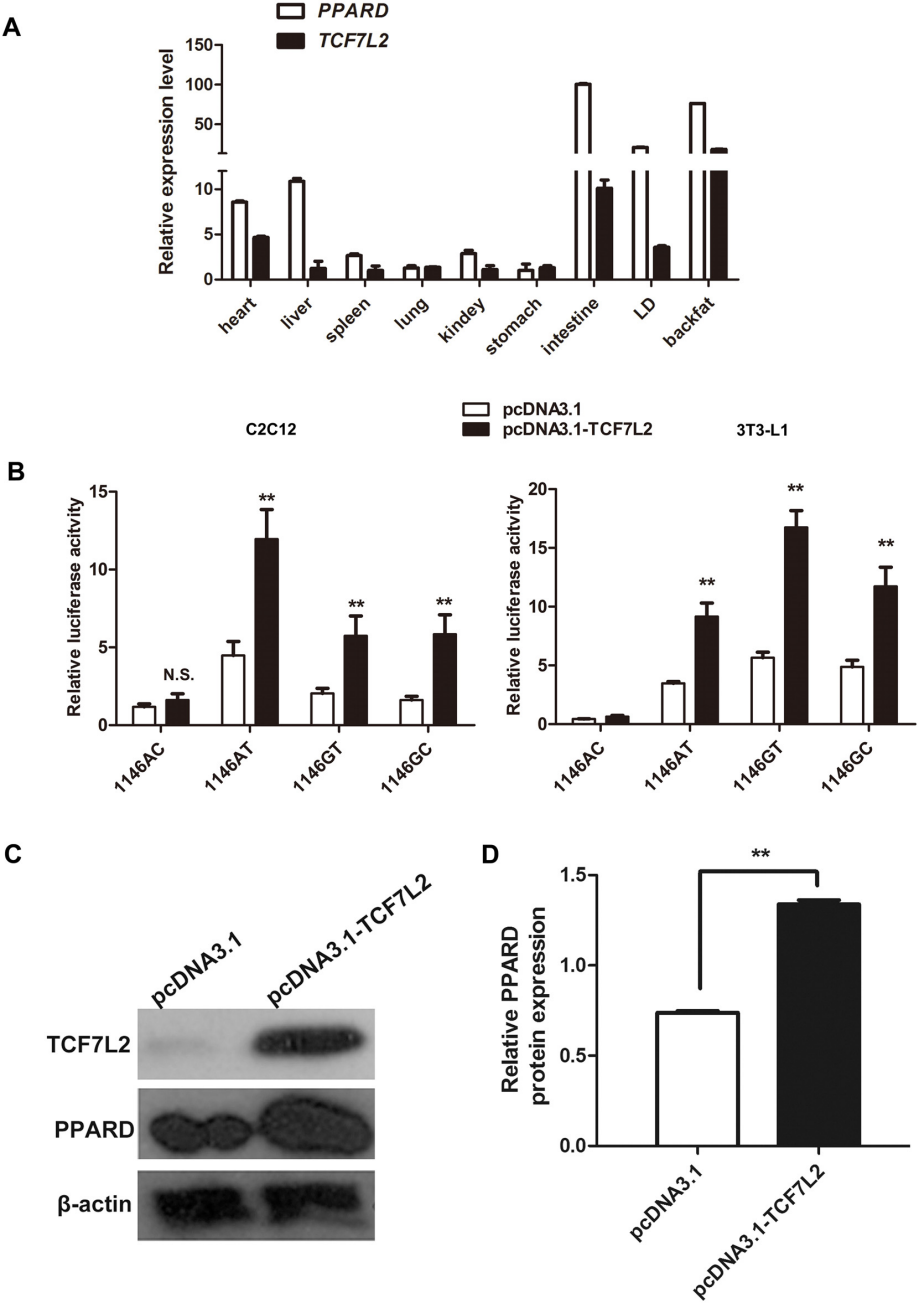
The SNPs in the 5’ regulatory region of *PPARD* gene affected transcriptional activation of TCF7L2. (A) The relative mRNA expression profiles of the porcine *PPARD* and *TCF7L2* gene in nine different tissues. (B) Transcriptional activities of four haplotypes determined by luciferase assay before and after *TCF7L2* overexpression in C2C12 myoblasts and 3T3-L1 cells. (C) The protein expression levels of *TCF7L2* and *PPARD* before and after *TCF7L2* overexpression in C2C12 myoblasts. C2C12 myoblasts were transfected with pcDNA3.1-TCF7L2 vector and empty vector, and then the total protein extracted after 48 h transfection. The protein levels of *TCF7L2* and *PPARD* genes were analyzed by western blotting. (D) Relative *PPARD* protein expression levels represented by ratio of detected protein to *β-actin* protein expression level after *TCF7L2* overexpression.

**Fig 5:**
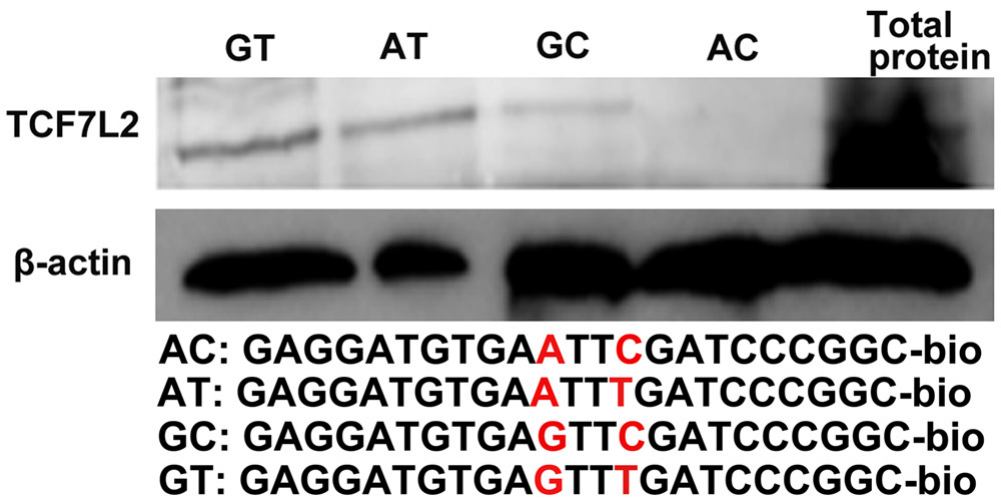
DNA pull-down results showing the interaction of TCF7L2 with the four haplotypes in 3T3-L1 cell *in vivo*. 3T3-L1 cells were seeded in 10-cm dishes for 48 h, harvested and pull-downed by antibodies. Lane GT, AT, GC, and AC: immunoprecipitation results with anti-TCF7L2 monoclonal antibody. Lane total protein: the analysis of total cell lysates before immunoprecipitation to verify expression of *TCF7L2*. The sequences of four probes were shown under the panel.

The binding motif of transcription factor TCF7L2 contains AC/GA/TTCAAAG [40], which is similar to the reverse sequence of the region harboring the haplotypes. In the present study, a classic motif of TCF7L2 was not identified in *PPARD* promoter, but a variant TCF7L2 binding sequences ccgggATCAAACtcaca was located at − 660/− 644 bp. TCF7L2 could bind to the promoter of *PPARD* and increase its expression in human colorectal cancer (CRC) cells [41]. Therefore, we hypothesized that SNPs alters transcriptional activities of the promoter through affecting the binding capacities of TCF7L2 to its binding sites. We initially studied the tissue expression patterns of *TCF7L2* and *PPARD* genes in ten tissues including heart, liver, spleen, lung, kindey, brain, stomach, intestine, LD, and backfat of the four-month-old Large White pigs. The tissue expression profiles of two genes were very similar. They were all broadly expressed in the ten tissues, and highly expressed in liver and adipose which are active in fat synthesis (Fig 4A). To investigate the effects of *TCF7L2* overexpression on the promoter transcriptional activities of *PPARD* gene, we constructed the eukaryotic overexpression vector of the porcine *TCF7L2* (pCDNA3.1-TCF7L2) and co-transfected with the promoter fragments carrying the four haplotypes into C2C12 myoblasts and 3T3-L1 cells. *TCF7L2* overexpression significantly improved the transcriptional activities of the promoter fragments with haplotypes AT, GT, and GC, whereas there were not significant effects with haplotype AC (Fig 4B). The protein expression levels of *PPARD* were also up-regulated by *TCF7L2* overexpression in C2C12 myoblasts (Fig 4C and 4D). The binding capacities of TCF7L2 to the promoter fragments carrying four haplotypes were further analyzed by DNA pulldown in 3T3-L1 cells. The binding capacities of the fragments carrying haplotypes AT, GT and GC to TCF7L2 were significantly stronger than those of haplotype AC, and the binding capacity of haplotypes GT was the strongest. These results were in agreement with the results of the luciferase assay (Fig 5).

### The SNP g.1015A>G was significantly associated with fat deposition traits in three pig populations

We used *EcoR*IPCR-RFLP to genotype the polymorphism g.1015A>G. Digestion of the PCR fragments with *EcoR*Irestriction enzyme generated one fragment (genotype *GG*, 568bp), two fragments (genotype *AA*, 314 bp + 254 bp) or three fragments (genotype *AG*, 568 + 314 + 254 bp) (S1 Fig). Allele frequency distributions of *EcoR*I-RFLP polymorphism in thirteen pig populations are shown in Table 1. This polymorphic SNP was segregated in the tested pig populations, most of which had high frequencies of allele *G* at this site. The association analysis of the genotypes with the fat deposition and carcass traits were conducted in three pig populations including the Large White × Meishan F_2_ pigs, France, and American Large White pigs. There were significant differences in the fat deposition and carcass traits among different genotypes in Large White × Meishan F_2_ populations. Pigs carrying genotype *GG* had higher BFT, RIBBF, LFP and IFR, but lower CL than the *AA* or *AG* animals (Table 2). Furthermore, significant additive effects were also observed for these traits (Table 2). The significant associations of g.1015 A>G with the corrected live backfat thickness were also found in France and American Large White pigs (Table 3). Taken together, we concluded that this functional SNP was significantly associated with fat deposition traits.

**Table 1:** Allele distribution of *g*.*1015 A>G* polymorphism in thirteen pig populations.

			Allele frequency	
Population	Location	number	*G*	*A*
Bamei	Gansu province	12	0.71	0.29
Erhualian	Jiangsu province	34	0.68	0.32
Exihei	Hubei province	16	0.69	0.31
Huainan	Henan province	27	0.78	0.22
Jianli	Hubei province	19	0.79	0.21
Meishan	Shanghai	44	0.52	0.48
Wannan	Anhui province	19	0.63	0.37
Yangxin	Hubei province	19	0.47	0.53
English Large White	Hubei province	31	0.82	0.18
American Large White	Zhejiang province	409	0.68	0.32
France Large White	Zhejiang province	710	0.89	0.11
Landrace	Hubei province	34	0.80	0.20
Large White × Meishan F_2_ pigs	Hubei province	274	0.56	0.44

**Table 2:** Association results of *g*.*1015 A>G* polymorphism with carcass traits in Large White × Meishan F_2_ pig population.

	Genotype			Additive effect	Dominant effect
Traits	AA	AG	GG	a	d
BFT1 (cm)	3.44±0.11^a^	3.48±0.05^a^	3.76±0.08^b^	0.16±0.07*	0.04±0.03
67RIBBF (cm)	2.70±0.09^a^	2.72±0.04^a^	2.94±0.07^b^	0.12±0.05*	0.04±0.03
ABT (cm)	2.35±0.08^a^	2.43±0.04^a^	2.62±0.06^b^	0.13±0.05*	0.02±0.02
BFT2 (cm)	1.91±0.08^c^	2.04±0.04^ac^	2.17±0.06^ab^	0.13±0.05*	-0.002±0.02
BFT3 (cm)	1.71±0.1	1.83±0.05	1.94±0.07	0.11±0.06	0.001±0.03
CL1 (cm)	92.40±0.67^a^	91.10±0.34^a^	89.80±0.50^b^	-1.23±0.40	-0.1±0.20
CL2 (cm)	78.20±0.5^A^	77.40±0.28^A^	75.60±0.40^B^	-1.27±0.34*	-0.26±0.18
RN	14.70±0.10	14.68±0.10	14.69±0.20	-0.007±0.06	0.01±0.03
LFP (kg)	0.66±0.03^c^	0.71±0.03^ac^	0.77±0.02^ab^	0.05±0.02*	0.007±0.01
IFR (%)	1.10±0.05^ac^	1.06±0.02^c^	1.18±0.04^ab^	0.04±0.03	0.03±0.02

BFT1: backfat thickness at shoulder; ABT: average backfat thickness; BFT2: backfat thickness at thorax-waist; BFT3: backfat thickness at buttock; 67RIBBF: backfat thickness between 6^th^ and 7^th^ ribs; RN: rib numbers; CL1: Carcass body length 1; CL2: Carcass body length 2; LFP: Leaf fat percentage; IFR: Internal fat rate. Data were shown as means ± S.D. (standard deviation). Different superscript small letters in one row indicate significance level at *P*<0.05; Different superscript large letters in one row indicate significance level at P<0.01; Asterisk (*) represents significance level at *P*<0.05.

**Table 3:** Association results of *g*.*1015 A>G* polymorphism with the corrected backfat thickness at 100 kg body weight in France and American Large White pigs.

	Genotype			Additive effect	Dominant effect
Population	AA	AG	GG	a	d
American Large White	9.13±0.21^Aa^	9.59±0.09^b^	9.85±0.09^Bc^	0.36±0.11	-0.06±0.07
France Large White		8.53±0.10^A^	8.87±0.05^B^		

### The SNPs could significantly affect the expression of *PPARD* gene *in vivo*

**Fig 6:**
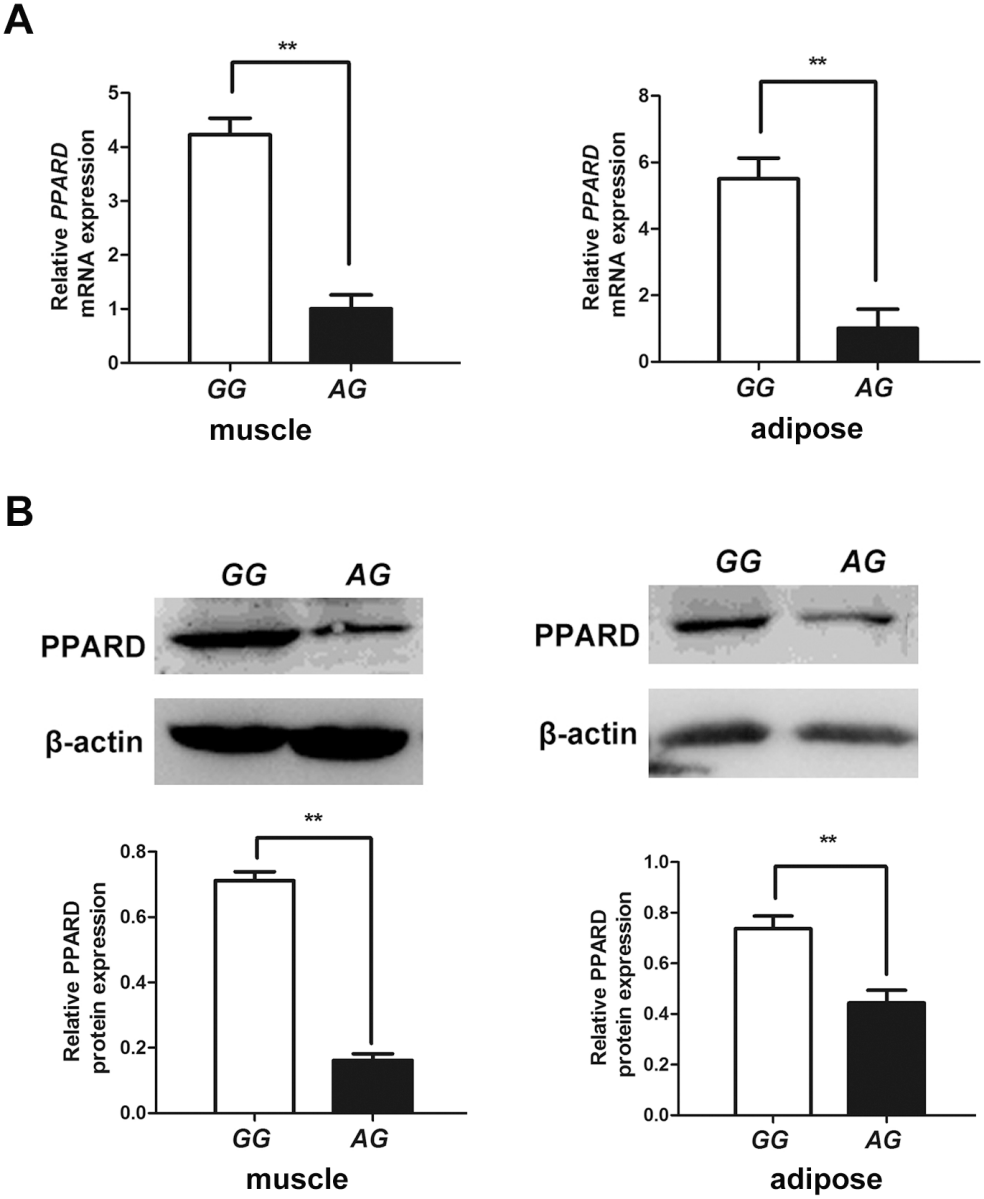
The SNPs significantly affect the expression of *PPARD* gene *in vivo*. (A) The relative mRNA expression levels of *PPARD* gene in muscle and adipose between genotype *AG* and *GG*. (B) The protein expression levels of *PPARD* gene in muscle and adipose between genotype *AG* and *GG*.

To estimate whether the SNP g.1015A>G could affect the gene expression *in vivo*, we selected France Large White pigs with genotypes *AG* and *GG* to compare the expression of *PPARD* gene in adipose and muscle tissues. The expression of *PPARD* gene between two genotypes was significantly different at both mRNA and protein level. Pigs with genotype *GG* had significant higher expression level than that of pigs with genotype *AG* (Fig 6).

## Discussion

Functional mutations in the regulatory region have effects on the gene expression and phenotypic variation. *PPARD* is a promising candidate gene for pig carcass traits [27, 33, 42]. Currently, there is not report about any functional SNPs in the 5’ regulatory region of the porcine *PPARD* gene. In this study, we identified the functional SNPs in the 5’ regulatory region which directly affected the promoter transcriptional activities. The transcriptional activity of the *PPARD* promoter fragment carrying haplotype AC was the lowest, and haplotype AC had the lowest binding capacities with TCF7L2. TCF7L2 is a member of the T cell factor (TCF) family of transcription factors, and involved in the control of cell growth and signaling of wingless-type MMTV integration site family (Wnt) receptors [43]. TCF7L2 interacts with β-catenin in the nucleus and regulates the expression of Wnt target genes by acting as transcriptional activator or repressor [44–47]. TCF4-binding sites are located at long distances from transcription start sites, and defined as evolutionarily conserved A-C/G-A/T-T-C-A-A-A-G motifs [40]. In our study, three TCF7L2 binding sites were predicted in the promoter region between nucleotides −1636 bp and + 464 bp, and one of the three sites was located within the promoter sequence harboring the two SNPs. TCF7L2 can bind to the promoter of *PPARD* gene and alter gene expression in human CRC cells [41]. We also confirmed that the expression of *PPARD* gene was up-regulated by *TCF7L2* overexpression in C2C12 cells. Besides, the porcine *TCF7L2* gene improved the transcriptional activities of the *PPARD* promoter fragments carrying the haplotypes AT, GT, and GC. However no significant effects on the haplotype AC were found, because there was no interaction between promoter fragments carrying haplotype AC and TCF7L2 protein.

*PPARD* gene has been implicated in lipid metabolism via several signaling pathways including Wnt/FZD and Wnt/β-catenin pathways. On the one hand, *PPARD* promote the accumulation of cholesterol and serum high density lipoprotein (HDL), and decrease the level of triglyceride in human macrophages by lipogenesis pathways such as Wnt/FZD and Wnt/β-catenin pathway [48, 49]. On the other hand, it reduces body weight, lipid droplet number and size, and up-regulates gene expression related to fatty acid oxidation [34]. In human population, three SNPs in the *PPARD* affect (lifestyle intervention) LI-induced changes in overall adiposity hepatic fat storage [50]. However, the molecular mechanism by which genetic variants within upstream regulatory region of *PPARD* gene affect fat deposition traits still remain unclear. In this study, the allele *A* was mostly linked with allele *C* at two loci in all the pig breeds studied, and no haplotype AT was found in American Large White pigs, France Large White pigs and Large White × Meishan F2 pigs studied. We also found that the functional variant (g. 1015A>G) had significant association with fat deposition traits in three pig populations and allele *A* was associated with lower fat deposition traits as well as lower gene expression of *PPARD in vitro* and *in vivo*. Previous studies showed that *PPARD* and *CDKN1A* gene in preadipocytes had lower expression levels in heterozygote MSQTL7/LWQTL7 (the heterozygous individuals carrying Meishan and Large White SSC7 QTL alleles) pigs than in homozygous LWQTL7/LWQTL7 (the homozygous individuals carrying Large White SSC7 QTL alleles) pigs at 28 and 150 days ages [51], which was in agreement with our results. Taken together, it can be inferred that haplotype AC inhibited the promoter transcriptional activity through decreasing the binding capacities of TCF7L2 to its binding sites, thereby repressed the *PPARD* expression and resulted in decreased fat deposition traits. Therefore, the expression change of *PPARD* gene may be an important cause of the major QTL effects for fat deposition.

In pig breeding schemes, the lean percentage in carcass can be indirectly selected by live backfat thickness because there is significant negative correlation between these two traits. In this study, there were significant differences in fat deposition traits between different genotypes. Pigs with genotypes *AA* or *AG* had significant lower fat deposition trait values compared with those of genotype *GG* in the pig populations studied. Considering the effects of allele *A* in this study, enhancing the allele frequency of allele *A* will lead to the reduction of backfat thickness in the pig populations of which the allele was not fixed at this locus. However, selection of allele *A* also has adverse effects on intramuscular fat and meat quality, as intramuscular fat and backfat deposition are negatively correlated.

In conclusion, our results provided a new evidence that the newly identified functional SNPs in the 5’ regulatory region of *PPARD* genes have significant effects on the fat deposition and carcass traits. Further work will be necessary to confirm the effects of this functional SNP in more pig populations and investigate the molecular mechanism regarding the roles of SNPs in phenotype variation.

## Supporting Information

S1 Fig Agarose gel electrophoresis showing the PCR-*EcoR*I-RFLP results of the porcine *PPARD* gene. Lane Marker: DNA molecular marker DL2000 (TaKaRa, Dalian, China); Lane 1, 2, and 6: genotype GG, 568 bp; Lane 3, 4: genotype AG, 568 bp + 314 bp + 254 bp; Lane 5: genotype AA, 314 bp + 254 bp.
(TIF)

S1 Table Primer information for DNA sequencing. (DOC)

S2 Table Primer information for the construction of pGL3-basic-based vectors. (DOC)

S3 Table Primer information for the expression profile analysis. (DOC)

S4 Table The predicted results of the binding capacity of transcription factors with different haplotype promoter fragments. (DOC)
