# Supplementary figures and images for: The Functional SNPs in the 5’ Regulatory Region of the Porcine PPARD Gene Have Significant Association with Fat Deposition Traits
Source: PLoS One. 2015 Nov 24;10(11):e0143734. doi: 10.1371/journal.pone.0143734 (PMC4658063; doi:10.1371/journal.pone.0143734)

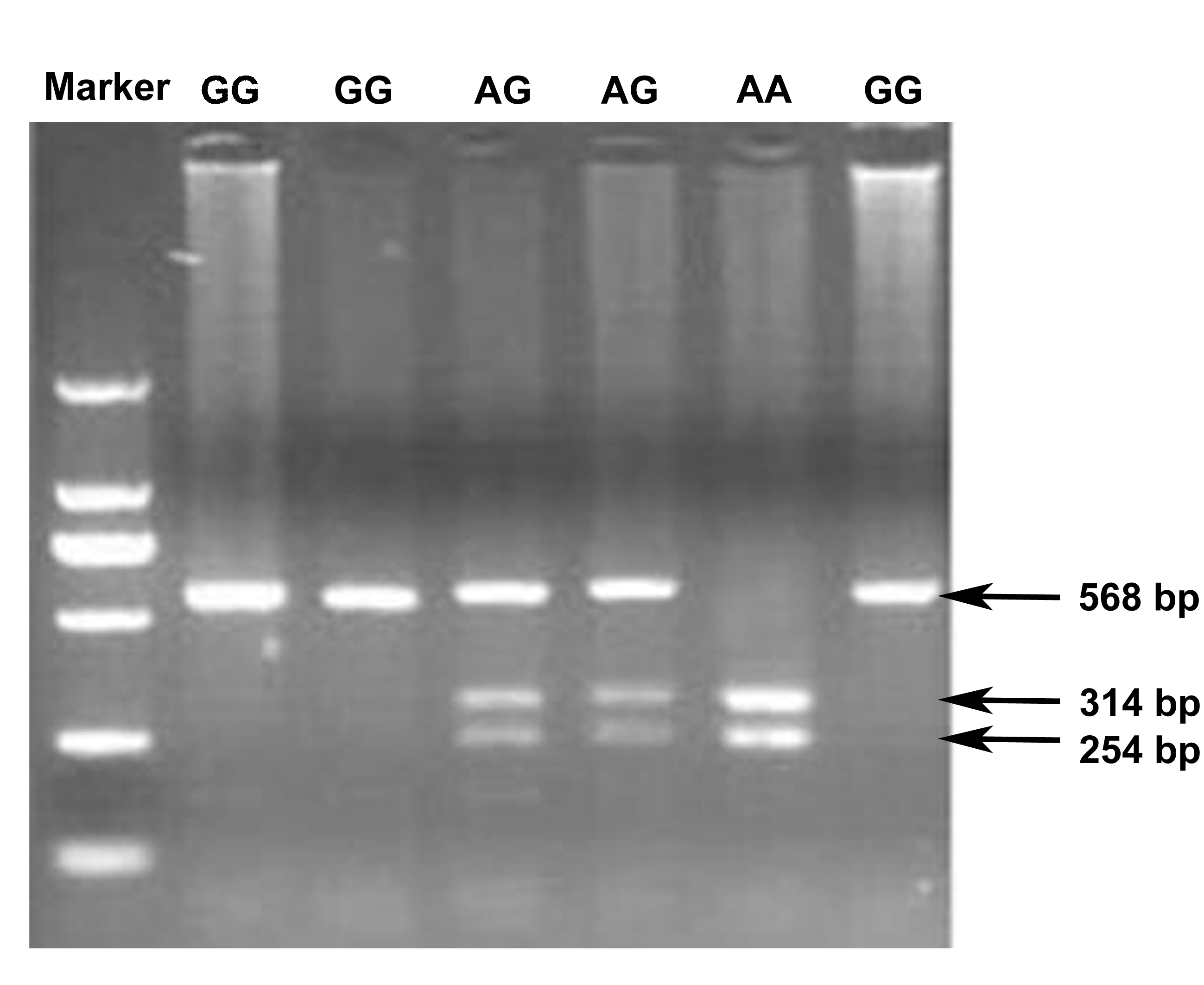

Supplement: S1 Fig — Lane Marker: DNA molecular marker DL2000 (TaKaRa, Dalian, China); Lane 1, 2, and 6: genotype GG, 568 bp; Lane 3, 4: genotype AG, 568 bp + 314 bp + 254 bp; Lane 5: genotype AA, 314 bp + 254 bp. (TIF) [file pone.0143734.s001.tif]
