# Supplementary material for: The Functional SNPs in the 5’ Regulatory Region of the Porcine PPARD Gene Have Significant Association with Fat Deposition Traits
Source: PLoS One. 2015 Nov 24;10(11):e0143734. doi: 10.1371/journal.pone.0143734 (PMC4658063; doi:10.1371/journal.pone.0143734)
Supplement: S1 Table — (DOC) [file pone.0143734.s002.doc]

**S1 Table. Primer information for DNA sequencing.**

| Primer | Sequence of primers (5’-3’) | Annealing temperature (°C) | length (bp) |
| --- | --- | --- | --- |
| *PPARD*-1F  *PPARD*-1R | TCAAAGGAGGGTACAGAAAG | 55.2 | 544 |
|  | AACCTCCTTGCCTTTGATA |  |  |
| *PPARD*-2F  *PPARD*-2R | TCAAAGGCAAGGAGGTTA | 56.6 | 568 |
|  | CTGGAGGAAACTGTGCTG |  |  |
| *PPARD*-3F  *PPARD*-3R | CCCGCCAAATCGAGGACA | 59.1 | 757 |
|  | TCCGCTGCTTGCCTATCC |  |  |
