# Supplementary material for: The Functional SNPs in the 5’ Regulatory Region of the Porcine PPARD Gene Have Significant Association with Fat Deposition Traits
Source: PLoS One. 2015 Nov 24;10(11):e0143734. doi: 10.1371/journal.pone.0143734 (PMC4658063; doi:10.1371/journal.pone.0143734)
Supplement: S2 Table — (DOC) [file pone.0143734.s003.doc]

**S2 Table. Primer information for the construction of pGL3-basic-based vectors.**

| Primer | Sequence of primers (5’-3’) | Annealing temperature (°C) | length (bp) | plasmid |
| --- | --- | --- | --- | --- |
| F1 | GGGGTACCGGCTGGAGCGCAAGGCCCG | 71.3 | 260 | pGL3-260 |
| F2 | GGGGTACCTCTCCAGAAGCTCGGTAA | 66 | 565 | pGL3-565 |
| F3 | GGGGTACCTTCTAGTTGCAGAAGCCTCA | 67.4 | 939 | pGL3-939 |
| F4 | GGGGTACCATTTTCTACAACAGTAT | 65 | 1032 | pGL3-1032 |
| F5 | GGGGTACCTCGCATGCTGTACCTGCC | 65 | 1077 | pGL3-1077 |
| F6 | GGGGTACCCACGACTAGTATCCAGGA | 65 | 1146 | pGL3-1146 |
| F7 | GGGGTACCGCGGTAACAAGCCCGACTAGATC | 66 | 1880 | pGL3-1880 |
| R | CGAGCTCGACGGAGTAAGAGGCCCAAAGAG |  |  |  |
