# Supplementary material for: The Functional SNPs in the 5’ Regulatory Region of the Porcine PPARD Gene Have Significant Association with Fat Deposition Traits
Source: PLoS One. 2015 Nov 24;10(11):e0143734. doi: 10.1371/journal.pone.0143734 (PMC4658063; doi:10.1371/journal.pone.0143734)
Supplement: S3 Table — (DOC) [file pone.0143734.s004.doc]

**S3 Table. Primer information for the expression profile analysis.**

| Gene | Primer | Sequence of primers (5’-3’) | Annealing temperature (°C) | length (bp) |
| --- | --- | --- | --- | --- |
| *PPARD* | *PPARD*-F | CAGAGCACTCGCTTCCCTC | 59 | 153 |
|  | *PPARD*-R | AGCCTGATGCCTTGTCCC |  |  |
| *TCF7L2* | *TCF7L2*-F | AATCCTTGCCTTTCACTTCC | 59 | 147 |
|  | *TCF7L2*-R | GCTGCCTTCACCTTGTATGT |  |  |
| *β-actin* | *β-actin*-F | CCAGGTCATCACCATCGG | 59 | 158 |
|  | *β-actin*-R | CCGTGTTGGCGTAGAGGT |  |  |
