# Supplementary material for: The Functional SNPs in the 5’ Regulatory Region of the Porcine PPARD Gene Have Significant Association with Fat Deposition Traits
Source: PLoS One. 2015 Nov 24;10(11):e0143734. doi: 10.1371/journal.pone.0143734 (PMC4658063; doi:10.1371/journal.pone.0143734)
Supplement: S4 Table — (DOC) [file pone.0143734.s005.doc]

**S4 Table.** The predicted results of the binding capacity of transcription factors with different haplotype promoter fragments

| haplotype | *ETS1* | *MEL1* | *Foxh1* | *POU2F1* | *TCF7L2* |
| --- | --- | --- | --- | --- | --- |
| AC | 0.922 | 0.950 | 0.941 | 0.869 | - |
| AT | 0.922 | 0.950 | 0.931 | 0.869 | 0.841 |
| GT | 0.922 | 0.950 | - | - | 0.843 |
| GC | 0.922 | 0.950 | 0.816 | - | - |

The sequence for analysis was atccaggaGGATgtga(a/g)tt(t/c)gATCCCGGC
